# Identification of the Patterns Produced in the Offensive Sequences That End in a Goal in European Futsal

**DOI:** 10.3389/fpsyg.2021.578332

**Published:** 2021-04-01

**Authors:** Mario Amatria, Javier Álvarez, Javier Ramírez, Víctor Murillo

**Affiliations:** ^1^Faculty of Educational Science, Pontifical University of Salamanca, Salamanca, Spain; ^2^Department of Physiatry and Nursing, University of Zaragoza, Zaragoza, Spain

**Keywords:** performance analysis, observational methodology, futsal, lag sequential analysis, goal

## Abstract

Victory is the ultimate aim in high performance sports; when it comes to team sports, the goal is the key that allows players to achieve that victory. This is the case with futsal which, due to its internal structure as well as the speed in the development of its game, makes the achievement of a goal not an isolated event, but rather more than one goal must be scored to achieve victory. The aim of the present study is to analyze the construction of offensive sequences that have resulted in goal-scoring in the two main European futsal leagues, the Spanish and the Italian, as well as to identify the patterns relating to offensive actions that have ended with a goal being scored. Observational methodology was used to develop the research and an *ad hoc* observation instrument (OAF-I) was developed for this purpose. The data was analyzed using inferential statistics as well as sequential analysis of delays in a diachronic analysis to identify the patterns of offensive actions. The results obtained enable recognition of a league’s idiosyncrasy patterns in goal-scoring in each of the leagues studied. The results obtained will allow experts to have a better understanding of how goals are scored and how to establish more specific training tasks, in both attack and defense.

## Introduction

The attack-defense duel is the essence of team sports (Garganta, 2009), where game systems are defined as the base structures representing the way in which players, as well as the functions to be developed, are distributed across the playing field, determining the offensive and defensive attitude of the players and the team (Pascual et al., 2019). That offensive or defensive attitude is extremely closely linked to the playing system used, a fact that gives rise to the team’s efficiency, which is improved by incorporating functions into the playing system (Mutti, 2003; Saraiva, 2010).

How effective a team is, in both collective sports in general and futsal in particular, is shown by the number of victories achieved (Amatria et al., 2019), which will ultimately depend on the final result of the match, obtained through the difference between goals scored and goals conceded, the goal being the most relevant indicator and predictor of performance in this sport (Schneider et al., 2015; Agras et al., 2016; Álvarez et al., 2018; Gómez et al., 2018).

As futsal is considered a low-scoring sport (Reina and Hernández-Mendo, 2012), the relevance of the goal as a performance indicator, as well as the analysis of the competition, acquire greater importance (Abdel-Hakim, 2014). This factor is especially admissible in regard to competition and high-level teams as the pressing professionalism and demand at every physiological, physical, technical-tactical, and psychological level increases equality amongst competitors (Álvarez et al., 2002, 2004).

For that reason, the study, analysis, and knowledge of the goal has generated a great deal scientific interest, despite the need for greater research into this sport (Cachón et al., 2012; Ramírez, 2015). The largest amount of research in that regard focuses on quantitative aspects (Hristovski et al., 2017), which is why the following questions arise: what circumstances need to take place in the development of the offensive action in order for the goal to be scored and what behaviors are repeated to achieve that? In this respect, the research community has shown growing interest in responding to how the offensive game develops and in trying to identify common behavioral patterns that are produced and replicated during particular attack sequences (Lapresa et al., 2013; Sarmento et al., 2016) in such a way that the repetition of behavior appears over time and favors their predictable recurrence (James, 2012).

Recognizing the behavioral patterns that occur in the development of the game and the means by which the goal is achieved (type of play, technical-tactical actions, etc.) is vital in optimizing the training process and in obtaining an advantage in competitive matches (James, 2012). For that reason, the determining aspect has begun to be valued, and there are several studies that provide relevant data and that are focused on analyzing offensive actions (Barbero, 2003; Irokawa et al., 2010; Travassos et al., 2011; Leite, 2012; Lapresa et al., 2013; Pascual et al., 2019). The following researchers focus on the world’s greatest leagues (Méndez et al., 2019), the Spanish and Italian leagues, which were the two greatest professional futsal leagues from 1996 to 2015 (Göral, 2018).

This study has a double objective: to analyze and identify the types of plays relating to the offensive sequences resulting in a goal in the two main European indoor football leagues, the Spanish and Italian leagues, during the 2014–2015 season; and to identify and compare behavior patterns relating to the offensive actions that result in a goal in both leagues during this competition.

## Materials and Methods

This study was carried out using observational methodology (Anguera, 1979), which found in the sport an ideal space for its implementation (O’Donoghue, 2009; McGarry et al., 2013; Anguera and Hernández-Mendo, 2015).

The design of the research has been carried out following the design of Anguera et al. (2011) and is of type N/D/M: nomothetic, datable (intra-sessional and inter-sessional), and multidimensional.

### Participants

To further develop our research, all of the goals scored in the 2014–2015 season from the two best professional futsal leagues in the 1996–2015 period (Göral, 2018) were taken as a sample (those being the Italian and Spanish leagues).

Videos of the goals were obtained by getting access to entire match footage, or to summaries in instances where footage could not be obtained from the various organizing bodies of the competitions, teams (the totality of matches corresponding to the Spanish league -240 matches-, and 83 to the Italian league), and open platforms (27 matches corresponding to the Italian league). At all times, entire match footage was taken as the first visual source; when such footage was unavailable, summaries were used.

The sample consisted of 5,145 multi-events, which gave rise to a total of 735 goals scored in the 110 matches that made up the regular Italian championship. The sample also consisted of 12,285 multi-events that gave rise to the 1,755 goals scored in 240 Spanish league matches, giving a total sample of 17,430 multi-events.

### Observational Instrument

The Observational Analysis of Futsal’s (OAF-I) observation instrument (Álvarez et al., in press) was used (see Table 1), being made up of the combination of category systems, meeting the requirements of exhaustive and mutually exclusive field formats (Anguera and Hernández-Mendo, 2013), the former being nested in the latter (Anguera et al., 2007).

**TABLE 1:** Observation instrument.

**Dimension**	**Category systems: codes and brief description**
Player no.	1, 2, 3, 4, 5, 6, …
League	ITA) Italian; SP) Spanish; RUS) Russian
Move	R1) 1st round of matches; R2) 2nd round of matches; R3) 3rd round of matches; R4) 4th round of matches; R5) 5th round of matches; R6) 6th round of matches; R7) 7th round of matches; R8) 8th round of matches; R9) 9th round of matches; R10) 10th round of matches; R11) 11th round of matches; R12) 12th round of matches; R13) 13th round of matches; R14) 14th round of matches; R15) 15th round of matches; R16) 16th round of matches; R17) 17th round of matches; R18) 18th round of matches; R19) 19th round of matches; R20) 20th round of matches; R21) 21st round of matches; R22) 22nd round of matches; R23) 23rd round of matches; R24) 24th round of matches; R25) 25th round of matches; R26) 26th round of matches; R27) 27th round of matches; R28) 28th round of matches; R29) 29th round of matches; R30) 30th round of matches
Round	RO1) round 1; RO2) round 2
Venue	LOC) local; AWA) away
Player position	GK) goalkeeper; WIN) winger; PIV) pivot; UNI) universal
Starting area	SA10) starting area 10; SA11) starting area 11; SA11a) starting area 11a; SA12) starting area 12; SA12a) starting area 12a; SA13) starting area 13; SA13a) starting area 13a; SA14) starting area 14—defensive areas -; SA20) starting area 20; SA21) starting area 21; SA22) starting area 22; SA23) starting area 23; SA24) starting area 24—creation area own pitch -, SA40) starting area 40, SA41) starting area 41; SA42) starting area 42; SA43) starting area 43; SA44) starting area 44—creation areas away pitch -; SA50) starting area 50; SA51) starting area 51; SA51a) starting area 51a; SA52) starting area 52; SA52a) starting area 52a; SA53) starting area 53; SA53a) starting area 53a; SA54) starting area 54—offensive areas -. View Figure 2
Ending area	EA10) ending area 10; EA11) ending area 11; EA11a) ending area 11a; EA12) ending area 12; EA12a) ending area 12a; EA13) ending area 13; EA13a) ending area 13a; EA14) ending area 14—defensive areas -; EA20) ending area 20; EA21) ending area 21; EA22) ending area 22; EA23) ending area 23; EA24) ending area 24—creation area own pitch -, EA40) ending area 40; EA41) ending area 41; EA42) ending area 42; EA43) ending area 43; EA44) ending area 44—creation areas away pitch -; EA50) ending area 50; EA51) ending area 51; EA51a) ending area 51a; EA52) ending area 52; EA52a) ending area 52a; EA53) ending area 53; EA53a) ending area 53a; EA54) ending area 54—offensive areas -. View Figure 2
Beginning	ROB) steal of the player; PI) Pass interception; PRS) pressure; CLEAR) clearance; MOV) move; REB) rebound; PLY) play; ANOT1) another; GK) goal kick; TI) throw in; CK) corner kick; DFK) direct free kick; IFK) indirect free kick; PE) penalty; DP) double penalty; MK) midfield kick; DB) dropped ball
Type of move	PA) positional attack (attack against organized defense developed in a sustained way); CA) counterattack (rapid attack after theft developed with the fewest possible passes and disorganized defense); CFG) counterattack in front of fly goalkeeper; PD) pressuring defense; FG) fly goalkeeper; SUP) superiority INF) inferiority: SP) Set pieces; ANOT2) another
Players on the pitch	PL1) 1 player; PL2) 2 players; PL3) 3 players; PL4) 4 players; PL5) 5 players
Number of passes	PS0) 0 passes; PS1) 1 pass; PS2) 2 passes; PS3) 3 passes; PS4) 4 passes; PS5) 5 passes; PS6) 6 passes; PS7) 7 passes; PS8) 8 passes; PS9) 9 passes; PS10) 10 passes; PS100) 11 or more passes
Ending of the game	DRB) dribbling; SR) short running; LR) long running; REB) rebound; DET) detour; 2FP) 2nd far post; OTP) one-two with pivot; OVL) overlap run; PM) pass to the midfield; OG) own goal; NON) none; ANOT3) another
Number of touches	TO1) 1 touch; TO2) 2 touches; TO3) 3 touches; TO4) 4 touches; TO5) 5 touches; TO6) 6 touches; TO7) 7 touches; TO8) 8 touches; TO9) 9 touches; TO10) 10 touches; TO100) 11 or more touches
Contact area	RIN) right instep; LIN) left instep; RINT) right interior; LINI) left interior; EXR) external right; EXL) left external; RT) right toecap; LT) Left toecap; RH) right heel; LH) left heel; HEAD) head; ANOT4) another
Penalty area	LS) low shot; RS) raised shot; HS) high shot
Laterality of the penalty area	RIGHT) right; LEFT) left; MIDD) middle
Time of goal	P11) 0′–5′ 1st half; P12) 6′–10′ 1st half; P13) 11′–15′ 1st half; P14) 16′–20′ 1st half; P21) 0′–5′ 2nd half; P22) 6′–10′ 2nd half; P23) 11–15′ 2nd half; P24) 16–20′ 2nd half.
Prior result	RV) victory; RT) draw-tie; RD) defeat
Temporary home scorer	It shows the temporary score: from 0 to 15, coinciding the value of the parameter with the number of goals scored by the home team.
Temporary away scorer	It shows the temporary score: from 0 to 15, coinciding the value of the parameter with the number of goals scored by the away team
Difference of goals	It shows the difference of goals between the teams, which means the goal scored. From 15 to -15, being 15 the maximum difference of goals in favor of the home team and -15, the maximum one in favor of the away team; 0 means draw
First result, halftime, and final result	FRFG) First result—first goal; FRFGRH) First result- first goal and result at the halftime; FRFGFHFR) First result—First goal, result at the halftime and final result; FGFRFR) First goal—First result and final result; RH) result at the halftime; RHFR) Result at the halftime and final result; FR) final result
Classification of round of matches	Classification with which the observed team begins the round of matches. From 1 to 16, being 1 the first position and so on
Final classification	Classification with which the observed team ends the league. From 1 to 16, 1 being the first position and so on

### Registration and Coding

**FIGURE 1:**
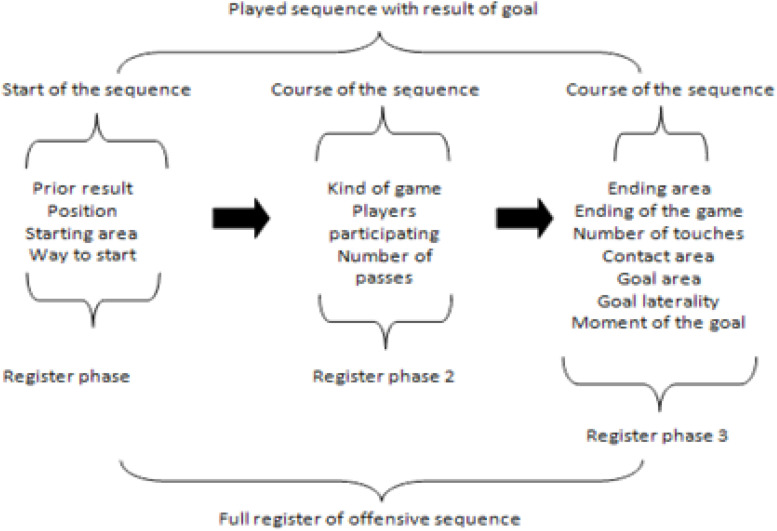
Structure of the recording of each play resulting in a goal: phases and dimensions to record.

The data was recorded using Lince software, version 1.2.1 (Gabin et al., 2012). The recording of each sequence was structured in three stages: a first stage, called observation, in which the offensive action was initially viewed in full without anything being noted; a second stage, in which the offensive action was viewed a second time along with the recording of the sequence itself, which, in turn, was carried out in three separate phases—the beginning of the offensive sequence, in which the categories corresponding to the Previous Result, Position, Starting Zone, and Starting Form dimensions were recorded; the development of the sequence, in which the categories corresponding to the Type of Play, Players involved, and Number of passes dimensions were recorded; and, finally, the end of the sequence, in which the categories corresponding to the rest of the dimensions were recorded (Figure 1). The last stage of the record consisted of viewing the action one last time to corroborate the data recorded in the previous stage. The recording unit ends when the team under observation scores the goal. The data analyzed are concurrent and event-based (Bakeman, 1978).

**FIGURE 2:**
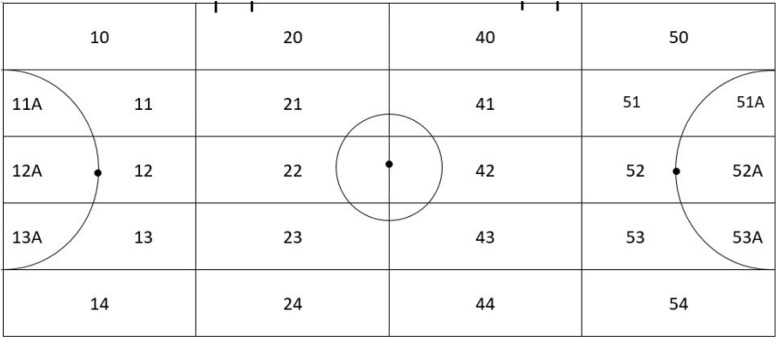
Field graph of spatial distribution according to the zones of the game.

According to Lapresa et al. (2013), Álvarez (2015), Amatria (2015), and Ramírez (2015) in futsal, and Casal et al. (2017) in soccer, the team under observation is interpreted as being in possession of the ball when at least one of the following three action situations occurs: (a) the player who receives the ball makes at least two contacts with it; (b) a player intercepts the ball and then a team mate continues the action; or (c) a player takes a throw-in in a set piece action.

### Data Reliability

The data was recorded by two observers, Physical Activity and Sports Sciences and National Futsal Coach graduates with more than 5 years’ experience in physical training and coaching in elite teams and observational methodology, who previously carried out a training process based on Anguera (2003). The first observer created data set 1 by recording samples on two occasions: the first, collecting all samples; and the second, collecting 10% of the total (inter-observer agreement). The second observer created a third record (intra-observer agreement) obtained on a consultative basis (Arana, 2011; Arana et al., 2016).

In order to determine the reliability (in the form of concordance) of the data obtained from the observation instrument, Cohen’s kappa coefficient (Cohen, 1960) was used throughout the GSEQ program (Bakeman and Quera, 2011), version 5.1. The Cohen’s kappa coefficient values corresponding to the data packages recorded by both observers have been calculated by dimensions or criteria of the observation instrument, giving a minimum value of κ> 0.9 and agreement >95% (Table 2), which corresponds, in line with the values determined by Landis and Koch (1977), to an *“almost perfect.”*

**TABLE 2:** Cohen’s Kappa results of first and second observer for each of the leagues analyzed, by dimensions.

	Spanish league	Italian league
Dimension	Kappa value	Kappa value
Player no.	1	1
League	1	1
Move	1	1
Round	1	1
Venue	1	1
Player position	0.96	0.92
Start area	0.9	0.93
Ending area	0.92	0.9
Beginning	0.91	0.95
Type of move	0.95	0.91
Players on the pitch	0.93	0.95
Number of passes	0.94	0.96
Ending of the game	0.91	0.93
Number of touches	0.97	0.98
Contact area	0.98	0.97
Penalty area	0.97	0.98
Laterality of the penalty area	0.91	0.96
Time of goal	1	1
Prior result	1	1
Temporary home scorer	1	1
Temporary away scorer	1	1
Difference of goals	1	1
First result, halftime, and final result	1	1
Classification of round of matches	1	1
Final classification	1	1

The data was analyzed using the two types of analysis that make up mixed methods, i.e., quantitative analysis and qualitative analysis (Anguera et al., 2014).

In order to respond to the first of these objectives and to analyze and identify the types of plays corresponding to the offensive sequences ending in goals, a crossover analysis between leagues was carried out, establishing three pairs of categorical variables that, due to their internal relationships, are relevant for the study. Firstly, the PA (positional attack) and CA (counterattack) pair have been established because they are attacks against structured and unstructured defenses. The second established pair comprises the FG (fly goalkeeper) and the CAFG (counterattack against the fly goalkeeper), in relation to the goals achieved during this particular facet of the sport. Finally, a third pair of relevant categories has been established, corresponding to the SP (set piece) category and to the remaining moves, as they are played standing in front of the those that occur in motion.

For the data analysis of those three pairs, analysis was done by searching for associative relationships between categorical variables. For this, the parametric Pearson chi-square test (χχ2) was adopted using the following formula (Carro et al., 1997), $\chi^{2}=\sum_{i=1}^{I}\sum_{j=1}^{J}\frac{{\left(f\,e_{i\,j}-f\,t_{i\,j}\right)}^{2}}{f\,t_{i\,j}}$, establishing a statistical significance relationship between variables analyzed when *p* < 0.05.

A diachronic analysis of the data was done at a qualitative level to meet the second objective of this study: identifying the goal pattern in the Spanish and Italian leagues. Specifically, sequential-delay analysis was carried out (Sackett, 1980), which allows for the identification of behavioral patterns understood as “behaviors that occur with greater cohesion than mere chance” (Anguera, 1990, p. 202).

To carry out the calculation, a behavior from within the categorical lag data was taken as the initial hypothesis (core behavior). The lag number indicates the order in which it occurs.

Once the conditional behaviors and lags of interest have been defined, a matching frequencies table based on the given behavior is generated (Sackett, 1987). That table can be used to observe the positive behaviors (when conditional probabilities are greater than the expected) and lay down every link in the behavior pattern at each lag (Anguera et al., in press).

This can be applied to any research situation in which categorical actions are measured in an ordered sequence of actions or time (Castellano, 2000). Doing so establishes the suitability of the technique for data analysis, since it makes use of the capacity of the observational methodology to capture behaviors that develop according to parameters of order and duration (Anguera et al., 2000). Using the GSEQ software, v 5.1 (Bakeman and Quera, 2011), the adjusted residuals have been calculated between the behavior criterion corresponding to the League dimension and the conditioned behaviors corresponding to the rest of the dimensions that make up the instrument (player position, play start zone, play end zone, play start form, type of play, players involved, number of passes, play end, number of touches, contact surface, goal zone, goal laterality, time of goal, and previous result). Conditioned behaviors have been analyzed prospectively in relation to the established criteria categories, from co-occurrence 0 to delay 6.

In order to test the existence of a statistical association between conditional and expected behavior, a binomial formula can be applied (Anguera, 1990):

(1) $$z=\frac{P_{O\,b\,s\,e\,r\,v\,e\,d}-P_{e\,x\,p\,e\,c\,t\,e\,d}}{\sigma_{e\,x\,p\,e\,c\,t\,e\,d}}$$

where

(2) $$\sigma_{e\,x\,p\,e\,c\,t\,e\,d}=\sqrt{\frac{P_{o\,b\,s\,e\,r\,v\,e\,d}(1-P_{e\,x\,p\,e\,c\,t\,e\,d}}{N_{C\,r\,i\,t}}}$$

and with N_*Crit.*_ being the total core behavior appearances.

According to Bakeman and Gottman (1987) and Arias-Pujol and Anguera (2020), transitions greater than 1.96 show a statistically significant relationship (*p* < 0.05) of activation between the given behavior and the target behavior; transitions smaller than −1.96 indicate a statistically significant relationship of inhibition between the behavior criterion and the conditioned behavior.

## Results

Presented below are the results obtained from analysis of the type of play in which the goal is scored during the competitions examined. For further clarification of the results, three analysis sections are presented, taking into account the typological pairs of established moves.

In Table 3, it can be seen how, of the 1,719 goals scored in the Spanish league during the 2014–2015 season, the types of play through which the goal is scored are instigated from positional attack (PA), with 27.7% of the total, followed by counterattack (CA), with 26.8%, and set piece (SP), with 24%. On the other hand, of the 695 goals scored in the Italian league in the same season, the highest percentage (26%) relates to set piece (SP), followed by 24.2% from counterattack plays and, finally, positional attack (PA), with 23.3%.

**TABLE 3:** Offensive actions that resulted in a goal according to the type of play in the Spanish and Italian leagues during the 2014–2015 season.

	Spanish league	Italian league	Total
PA	482 (27.7%)	168 (23.3%)	650
CA	465 (26.8%)	174 (24.2%)	639
CFG	126 (7.0%)	60 (7.8%)	186
PD	125 (7.0%)	47 (5.9%)	172
FG	110 (6.1%)	83 (11.1%)	193
SUP	19 (1.0%)	17 (1.7%)	39
INF	1 (0.1%)	0 (0.0%)	1
SP	424 (24.4%)	186 (26.0%)	610
Total	1,755 (100%)	735 (100%)	2,490

Table 4 shows the results obtained by the chi-squared test corresponding to the first pair of established moves (PA-CA), where no significant results are seen between those categories. Considering the analysis of the chi-squared tests corresponding to the second established pair (FG-CAFG), Table 4 shows the results obtained where significant differences have been detected between those variables. Finally, Table 4 presents the results from the analysis of the last established relational pair of categories (SP—rest of plays), where no significant differences have been found between them.

**TABLE 4:** Results of the chi-squared test of the relationships between variables in both leagues.

	Value	df	Asymp. Sig. (bilateral)	*N* of valid cases
PA-CA	0.304	1	0.582	1,266
FG-CAFG	5.036	1	0.025*	357
SP- rest of plays	0.706	1	0.401	2,457

*Significant results.

At a qualitative level, Tables 5, 6 present the results obtained through sequential-lag analysis of the Spanish and Italian leagues.

**TABLE 5:** Significantly adjusted remainder that reflects sequential patterns of offensive actions leading to a goal in the Spanish futsal league.

	Lag 0	Lag 1	Lag 2	Lag 3	Lag 4	Lag 5	Lag 6
Position		WIN (18.5)					
Starting area		SA22 (3.18) SA23 (3.18) SA42 (5.57) SA43 (2.9)					
Ending area			EA11a (2.79) EA13a (2.57) EA43 (2.84) EA52 (4.21)				
Beginning			SP (2.42). CLEAR (4.26). REB (3.03). DFK (3.2)				
Type of move			CA (2.57)				
Players on the pitch				PL3 (3.8)			
Number of passes				PS2 (4.51)			
Ending of the game				OVL (5.43) ANOT3 (19.56)			
Number of touches					TO1 (5.22)		
Contact area					RIN (3.99) LIN (3.55)		
Penalty area						RS (3.63)	
Laterality of the penalty area						MIDD (12.76)	
Time of goal							P13 (2.24)
Prior result							RV (2.51)

*Significant adjusted residuals of the lag sequential analysis for league.*

**TABLE 6:** Significantly adjusted remainder that reflects sequential patterns of offensive actions leading to a goal in the Italian futsal league.

	Lag 0	Lag 1	Lag 2	Lag 3	Lag 4	Lag 5	Lag 6
Position		WIN (3.52). UNI (23.49) INDEF (5.7)					
Starting area		SA22 (3.18). SA23 (3.18) SA42 (5.57). SA43 (2.9)					
Ending area			EA20 (2.73). EA50 (2.88)				
Beginning			SP (4.03). PRS (2.44)				
Kind of move			FG (3.74)				
Players on the pitch				PL4 (2.75)			
Number of passes				PS3 (4.33)			
Ending of the game				PM (2.66)			
Number of touches					TO2 (2.75)		
Contact area					LIN (3.06). RT (3.16) LT (2.82)		
Penalty area						LS (2.57)	
Laterality of the penalty area						LEFT (2.13)	
Time of goal							P10 (2.44) P20 (5.7)
Prior result							RD (2.06)

*Significant adjusted residuals of the lag sequential analysis for league.*

The goal-scoring pattern established in the Spanish league shows the player who occupies the winger position as the author of the goal, originating from offensive-action zones 22, 23, 42, and 43, generated by robbery (ROB), clearance (CLEAR), rebound (REB), or through a direct free kick (DFK), and ending in zones 43 and 52. The type of play that manifests itself is the counterattack (CA), in which three players intervene (P3) and two passes are used (PS2), finishing tactically by means of splits or overlaps (OVL) or other unstructured tactical alternatives (OTR2). The technical action by which the goal is scored consists of a single touch (TO1) executed with the inside of the foot (RIN, LIN), with an elevated trajectory (RS) and centered on goal (MIDD), occurring between minutes 11:00 and 15:59 of the first part (P13) with a favorable result (RV).

For its part, the Italian league presents a goal-scoring pattern in which the player who scores the goal occupies the winger and universal positions, having originated their offensive action from zones 22, 23, 42, and 43, generated by robbery (ROB) or pressure (PRS) and ending in zone 50. The type of play displayed is one in which the fly goalkeeper (FG) is used, in which four players (P4) intervene and three passes are used (PS3), finishing tactically with a pass to the center or the midfield (PM). The technical action by which the goal is scored consists of two touches (TO2) executed in the last instance, that is, the last contact with the ball, with the instep of the left foot (LIN) or with the toe, both right (RT) and left (LT), with a flat trajectory (LS) and directed to the left of the goal (LEFT), occurring both in the first period of the first (P10) and second half of the match (P20) with a tie result (RT).

## Discussion

This study was proposed with two complementary objectives: firstly, to analyze and identify the types of plays corresponding to the offensive sequences resulting in goals in the two greatest European futsal leagues, the Spanish and Italian leagues, during the 2014-2015 season, and secondly, to identify and compare the behavioral patterns corresponding to their offensive actions that resulted in a goal in both leagues during that competition.

With reference to the first of these objectives, the results show that in the Spanish league, the highest number of goals (27.7%) was achieved through positional attack, which indicates that the action is carried out against a structured defense that is properly positioned, compared with 23.3% reached by the Italian league in this type of attack. Those results are consistent with those obtained by Álvarez et al. (2004) and Méndez et al. (2019), whose studies recognize that most of the goals scored occur through positional attacks.

With respect to the achievement of the goals through counterattack, as well as set piece actions, the results of both leagues are in close proximity to each other, resulting in 26.8 and 24.2% in the Spanish and Italian leagues, respectively, taking into account the first type, and 24.4% in the Spanish league and 26% in the Italian league with reference to the second, set pieces. Those results agree with the ones obtained by Álvarez et al. (2018) and Pascual et al. (2019), who determined that goals in futsal matches are produced through quick plays. In that respect, Álvarez et al. (2018) obtained differences between two seasons. In the 2012–2013 season, goals through positional attack and counterattack were very similar, with values higher than those of this study in both cases; in the 2013–2014 season, results were obtained that were very close to the ones presented in terms of the goals scored in counterattack, but much higher in those scored in positional attack, which shows that in the remaining actions, such as set pieces or fly goalkeeper, the goals scored were fewer than those obtained in our study.

In the Italian league, 11.1% of goals are scored using the goalkeeper-player, compared with 6.1% in the Spanish league. By account of the counterattack against the fly goalkeeper, referring to the plays in which the ball is recovered when that situation is being defended and a goal is scored immediately thereafter, goals in counterattack would exceed those scored in positional attack in both leagues, 33.8% in the Spanish league and 32% in the Italian league, a figure that demonstrates the effectiveness of the plays in which the ball is recovered with a disorderly rival defense and the relevance reached by the transition phases (Jones et al., 2004) in the success of the offensive action.

As regards the second objective, two types of clearly differentiated patterns are identified that reveal the difference in how the two leagues score their goals, as has been set out in section “Results.” However, when comparing the two goal-scoring patterns, we find several variants between them.

Firstly, by focusing on the player who scores the goal and his technical execution in doing so, we see how in the Spanish league the winger is the player that appears to be statistically significant, while in the Italian league we find that the goal is scored by the player who occupies the wing position but is predominantly marked as the universal player; in that instance, and unlike the Spanish league, the demarcation is exclusive to the Italian league, identifying the players who occupy that position as those who can play in any of the other three field positions (wing, pivot, and defender) (Álvarez et al., 2020).

Regarding the technical execution developed, the results show that the goal in the Spanish league is scored with a prior touch: the player controls the ball until the final hit, for which he makes use of the inside of his foot, seeking safety and precision. However, in the Italian league, the goal is scored with two prior touches, using the left instep or the toe; it seems clear that the player who finishes has time to prepare the shot, placing the ball well and striking with power, as shown by the surfaces used. Those results are diametrically opposed, those of the Spanish league matching those obtained by Pascual et al. (2019), which show that the surface most used to score a goal is the inside of the foot, and matching the results of the Italian league found by Lapresa et al. (2013), which show that the majority of goals are achieved via instep. This shows that there is no consensus as to the optimal hitting surface. On the other hand, considering the exact area where the ball enters the goal, the central space and half height are identified, which shows that when the player hits, the goalkeeper is out of position and cannot stop a ball that enters through the center of the goal. Those results are contrary to the ones obtained in the Italian league and by other studies, which all show that the bulk of goals come flat through the goal (Martín, 2009; Álvarez et al., 2018; Pascual et al., 2019).

Regarding the area in which those successful actions occur, the behavior patterns identified in both leagues coincide with the starting zones of play (SA22, SA23, SA42, and SA43), although their completion occurs in different areas, those being the border areas with the rival field (EA52) in the case of the Spanish league and in the areas belonging to the left lane, both in the player’s field (EA20) and in their rival field (EA50), which are characteristic zones used by defensive teams to avoid completions by using the touchline to reduce the action space available to attacking players. Studies such as that of Lapresa et al. (2013); Álvarez et al. (2018), and Pascual et al. (2019) in respect of the end zone agree with the results obtained in the Spanish league. In addition to those area zones, this study, with reference to the Spanish league, shows the appearance of significant finishing zones in the player’s own field (EA11a and EA13a), which relates to goals produced before attacks with the goalkeeper, who accounts for 6.1% of goals.

Based on the start of play, a pattern is established in the Spanish league where the action that reaches the goal begins after clearance or theft of the ball from the player, which means that the subsequent play is a counterattack in which three players participate, making two passes in the development of the action and a split as a technical-tactical element prior to the goal. In the Italian league, the start of play occurs after theft, as is the case most of the time in the Spanish league. However, the counterattack is not the standard type of play, but the favorable use of the fly goalkeeper, in which four players participate and there are three prior passes with a pass to the center as a technical-tactical action prior to the goal. Those results show a characteristic goal pattern at a qualitative level with a recovery of possession in central areas after theft of the ball, after which, in the Spanish league, there is a quick counterattack play to finish near the rival goal. Conversely, the Italian league prefers to adopt the approach of ensuring possession with the use of the fly goalkeeper through numerical superiority, not to look for a quick play that could cause a loss after recovery. Those results reveal two clear styles of play that predominate in each competition, being more elaborate in the Spanish league and more conservative in the Italian league.

Finally, with reference to the moment of the goal being scored and the previous result, the qualitative results show that, in the Spanish league, goals are scored from the 10th to the 15th min of the first half with a prior favorable result. That is a result contrary to the one obtained by Pascual et al. (2019), which shows that goals occur in the last 10 min of the game. In the Italian league, the results obtained do not establish differences between the two parties and they do not specify the result prior to the goal.

## Conclusion

Throughout this study, we have given a response to the established objectives set out in the research. Firstly, the typology of the moves that result in a goal of the two best European futsal leagues have been analyzed and identified, the counterattack being the type that stands out for its reach in the Spanish league, while set pieces appear to be preferred in the Italian league.

As for the second of the objectives set out in this research, two behavioral patterns have been identified for goal-scoring that strengthen the previous conclusion, but in this case from a qualitative perspective, where they show the manner in which goals are scored.

In the case of the Spanish league, the winger’s territory is clearly defined by a counterattack action that takes place in the middle zones of the field and in which two players intervene, scoring the goal through splits and technical executions, which is simple (by means of a single touch) yet complex (due to the direction and precision of where the ball enters the center top region of the goal).

As for the Italian league, a pattern of behavior is revealed with the following structure: the player who occupies the universal position scores a goal, initiating the offensive action in central areas of the own field and the rival field after theft, and finishing it from the left lane both from the own field and the rival field, the usual type of play being the favorable use of the fly goalkeeper, in which four players participate and there are three prior passes, with a pass to the center as a technical-tactical action prior to the goal. The execution of the shot to goal is composed of two touches using the left instep or the toe, and the ball enters the goal flat.

In light of the results obtained, it can be inferred that scoring a goal in the Spanish league is most effective in developing offensive transitions, which lead to a greater complexity and speed of the game; this, in turn, demands a higher standard of players and teams, both technically and tactically. Conversely, in the Italian league, set-piece actions are the first option to achieve the goal, which highlights the relevance of that type of action in a match, as well as the differences in the game between the two best European leagues of the last decade.

## Practical Applications

The results obtained will enable technicians to gain a better understanding of how goals are scored and will allow them to set more specific training tasks, in both attack and defense. Thanks to established patterns, tasks can be conditioned through the type of defense or attack to be used, determining, in a pre-established and priority-based manner, how, when, and where to end the move.

## Data Availability Statement

The raw data supporting the conclusions of this article will be made available by the authors, without undue reservation.

## Author Contributions

All authors have participated in the preparation and layout of the manuscript.

## Conflict of Interest

The authors declare that the research was conducted in the absence of any commercial or financial relationships that could be construed as a potential conflict of interest.
